# Standardised Framework for Quantitative Analysis of Fibrillation Dynamics

**DOI:** 10.1038/s41598-019-52976-y

**Published:** 2019-11-13

**Authors:** Xinyang Li, Caroline H. Roney, Balvinder S. Handa, Rasheda A. Chowdhury, Steven A. Niederer, Nicholas S. Peters, Fu Siong Ng

**Affiliations:** 1 National Heart and Lung Institute, Hammersmith Campus, Imperial College London, 72 Du Cane Rd, London, W120UQ UK; 2 School of Biomedical Engineering & Imaging Sciences, King’s College London, St. Thomas’ Hospital, Westminster Bridge Road, London, UK

**Keywords:** Cardiology, Medical research

## Abstract

The analysis of complex mechanisms underlying ventricular fibrillation (VF) and atrial fibrillation (AF) requires sophisticated tools for studying spatio-temporal action potential (AP) propagation dynamics. However, fibrillation analysis tools are often custom-made or proprietary, and vary between research groups. With no optimal standardised framework for analysis, results from different studies have led to disparate findings. Given the technical gap, here we present a comprehensive framework and set of principles for quantifying properties of wavefront dynamics in phase-processed data recorded during myocardial fibrillation with potentiometric dyes. Phase transformation of the fibrillatory data is particularly useful for identifying self-perpetuating spiral waves or rotational drivers (RDs) rotating around a phase singularity (PS). RDs have been implicated in sustaining fibrillation, and thus accurate localisation and quantification of RDs is crucial for understanding specific fibrillatory mechanisms. In this work, we assess how variation of analysis parameters and thresholds in the tracking of PSs and quantification of RDs could result in different interpretations of the underlying fibrillation mechanism. These techniques have been described and applied to experimental AF and VF data, and AF simulations, and examples are provided from each of these data sets to demonstrate the range of fibrillatory behaviours and adaptability of these tools. The presented methodologies are available as an open source software and offer an off-the-shelf research toolkit for quantifying and analysing fibrillatory mechanisms.

## Introduction

Both ventricular fibrillation (VF) and atrial fibrillation (AF) are complex cardiac arrhythmias, the prevalence of which are increasing rapidly with the ageing population^1^. Optical mapping has become an increasingly important pre-clinical tool for investigating the underlying mechanisms of fibrillation. *Ex-vivo* optical mapping of transmembrane voltage during AF and VF have demonstrated the existence of regions of functional reentry or rotational driver sites (RD), defined as regions where propagating wavefronts perpetuate around a phase singularity (PS)^2^. Phase singularities are defined as a point devoid of a definite phase while its neighbouring sites exhibit phases that change continuously. Stable wave reentry and rotational drivers are hypothesised to be responsible for the initiation and perpetuation of fibrillation^3^.

There is currently no standardised framework for processing fibrillation data. Previous studies have used different methodologies for processing optical mapping data during paced rhythm^4^, and for phase mapping^5–8^. Clinical studies focused on localising areas harbouring RDs have often relied upon qualitative visual analysis of phase maps or videos in fibrillation^9,10^. However, fibrillatory mechanisms and RD activity can demonstrate spatio-temporal fluctuations over time^11^, and tracking or quantifying these visually are labour-intensive, at times unfeasible and prone to a number of pitfalls that can lead to misinterpretation of the data. Certain methodologies exist for quantifying arrhythmia features from phase mapping^12–14^; however, these methods are technically challenging to implement. The diversity of methods used for analysing fibrillation data have in part led to conflicting interpretation of fibrillation data and a lack of agreement on the mechanisms that sustain AF and VF^15^.

**Figure 1:**
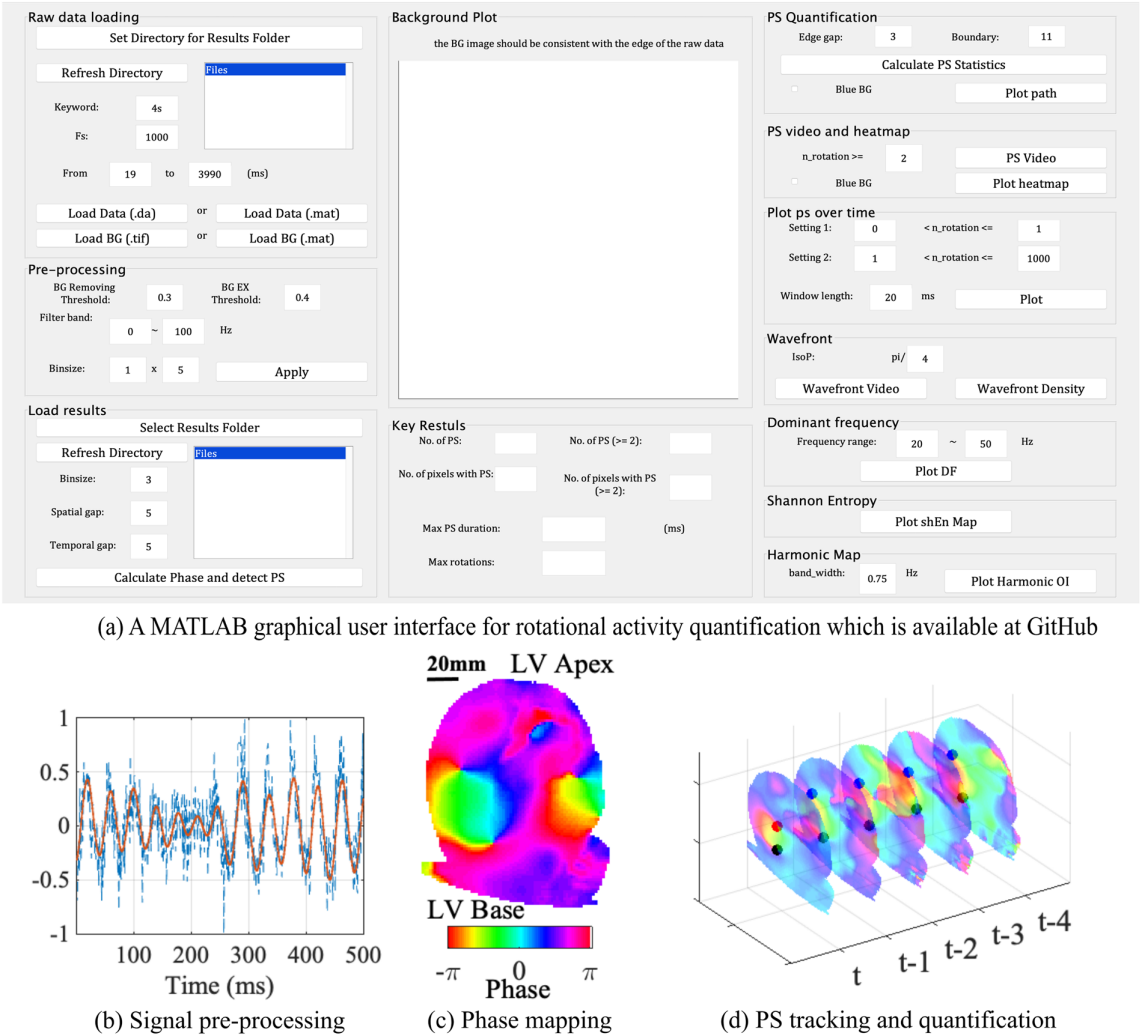
Quantification of rotational activity with fluorescence data. (**a**) The MATLAB graphical user interface for rotational activity quantification, (**b**) signal pre-processing, (**c**) phase mapping, (**d**) PS tracking and quantification.

To address and overcome this issue, in this work, we aimed to assess how the different analysis parameters and threshold settings can affect the conclusions drawn about the underling fibrillation mechanism. We also present a framework for analysis of fibrillation dynamics, and illustrate this using a range of fibrillation activity and experimental models, including optical fluorescence data from rat ventricular fibrillation (VF), canine atrial fibrillation (AF) and also simulation data. To make this work self-contained, we also included methodology details for pre-processing of optical mapping data and phase mapping. These proposed methods have been implemented and made available using a free open source customisable MATLAB-based analysis toolbox (available at github.com/xli15/optical-mapping-GUI), accompanied by a graphical user interface (GUI, Fig. 1a), with which automatic data processing for large data sets of fibrillation data can be achieved without intensive user input.

## Methods

### Pre-processing of optical fluorescence data

Optical mapping data can be noisy and thus a sequence of spatial and temporal filters was applied to process the signals before phase was calculated, following steps outlined by Laughner and Ng *et al*.^4^.

Figure 1(b) illustrates the effects of these pre-processing steps, and the aforementioned filtering functions have all been implemented, and are provided, in our toolbox.

### Phase mapping

Phase mapping transforms optical mapping transmembrane voltage data to instantaneous phase values. By depicting progression of the action potentials in a unified −*π* to *π* interval, phase mapping allows for the analysis of cardiac activation from signals with varying amplitudes and periodicity, such as in myocardial fibrillation. An example of a phase map is shown in Fig. 1(c). Details for pre-processing of optical fluorescence signals and parameter settings can be found in the Supplement, Pre-processing of Optical Fluorescence Data and Phase Mapping. Phase maps were post-processed to identify candidate PS locations using a PS detection technique, again previously described^16^. Example PS detections are shown in Fig. 1(d). Functions for both phase mapping and PS detection are available in our toolbox.

### Quantification of rotational driver

Once the fibrillatory data has been processed into phase data, accurate identification and tagging of PSs from propagating wavefronts, whilst challenging, is critical for accurate characterisation and quantification of RD dynamics. This step is often performed subjectively via visual assessment, which can lead to high inter- and intra-observer variability. The methodology for automated RD characterisation and how alteration of certain processing parameters can influence the results, is described below.

#### PS tracking

**Figure 2:**
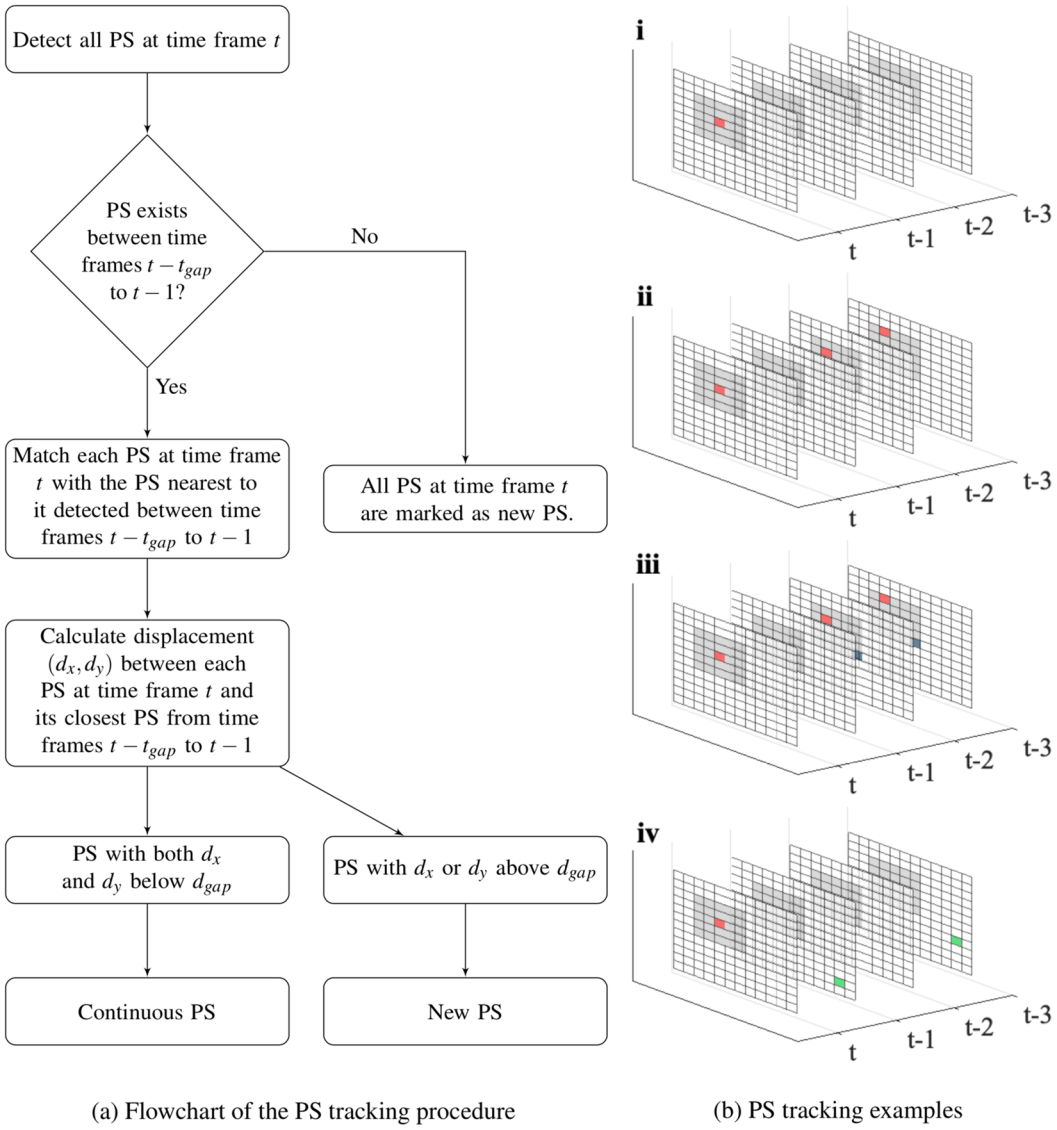
PS are tracked over time given pre-set spatial and temporal gaps, i.e., *d*_*gap*_ and *t*_*gap*_. (**a**) shows the PS tracking flow chart. (**b**) shows four scenarios for tracking, where *d*_*gap*_ = 3, *t*_*gap*_ = 3, and the PS at *t* is marked as a red dot. In (**b**-**i)**, no PS are detected in the last 3 time frames, and so the PS at *t* is classified as a new PS. In (**b**-**ii)**, although there is a missing PS detection at *t* − 1, there is a PS within *d*_*gap*_ at *t* − 2 and *t* − 3, and so the PS at *t* is classified as a continuous PS. In (**b**-**iii)**, there exists more than one PS at *t* − 1 and *t* − 2 (in red and blue), and the red PS at *t* − 1 is spatially closest to the PS at *t*. In (**b**-**iv)**, all the PS detected over the last 3 time frames are outside the grey area. Therefore, the PS at *t* is classified as a new PS.

To quantify key properties of rotational activity in fibrillation, PSs that exhibit continuous rotational activity were identified using our proposed PS tracking technique^11,17^. Even the highest quality optical mapping data may be prone to noise in real-world conditions and PS detections may be missed, and even the most stable RDs can shift spatially across different pixels. To track stable continuous PSs separate from de-novo PS occurring in close proximity to the trajectory of an existing PS in neighbouring time frames, an acceptable allowance needs to be made for spatial displacement, termed *d*_*gap*_, and temporal displacement, termed *t*_*gap*_. The procedure of PS tracking is illustrated in Fig. 2.

A given PS tracked in a current time frame is considered continuous and attributed as belonging to the trajectory of PSs in previous frames if it occurs within the defined *d*_*gap*_ and *t*_*gap*_. For example, Fig. 2(b) shows examples where a PS in the current time frame is tracked using the previous 3 time frames, i.e., *t*_*gap*_ = 3. The grey area marks a spatial allowance of *d*_*gap*_ = 3 pixels in the *x* and *y* plane. In this example, the PSs in the defined *t*_*gap*_ frames that are closest to the PS at the current frame are added to the existed trajectory of the PS. The PS is considered to be de-novo in the case that there are no PS detected in the last 3 frames (Fig. 2(b)-i) or if all detected PS in the last 3 frames are outside of the grey area determined by *d*_*gap*_ (Fig. 2(b)-iv). As such, *d*_*gap*_ controls the sensitivity of classifying a PS as new or propagated. If *d*_*gap*_ is small, PSs are more likely to be classified as new PSs; while if *d*_*gap*_ is large, PSs are more likely to be assigned to existing PS trajectories as a continuous PS.

#### Calculating the number of rotations

Accurate estimation of the number of rotations for a RD relies upon the identification of the leading edge of the propagating wavefront and calculation of the distances between the edges, and these steps could be sensitive to the threshold set for the length of the wavefront from the PS point.

**Figure 3:**
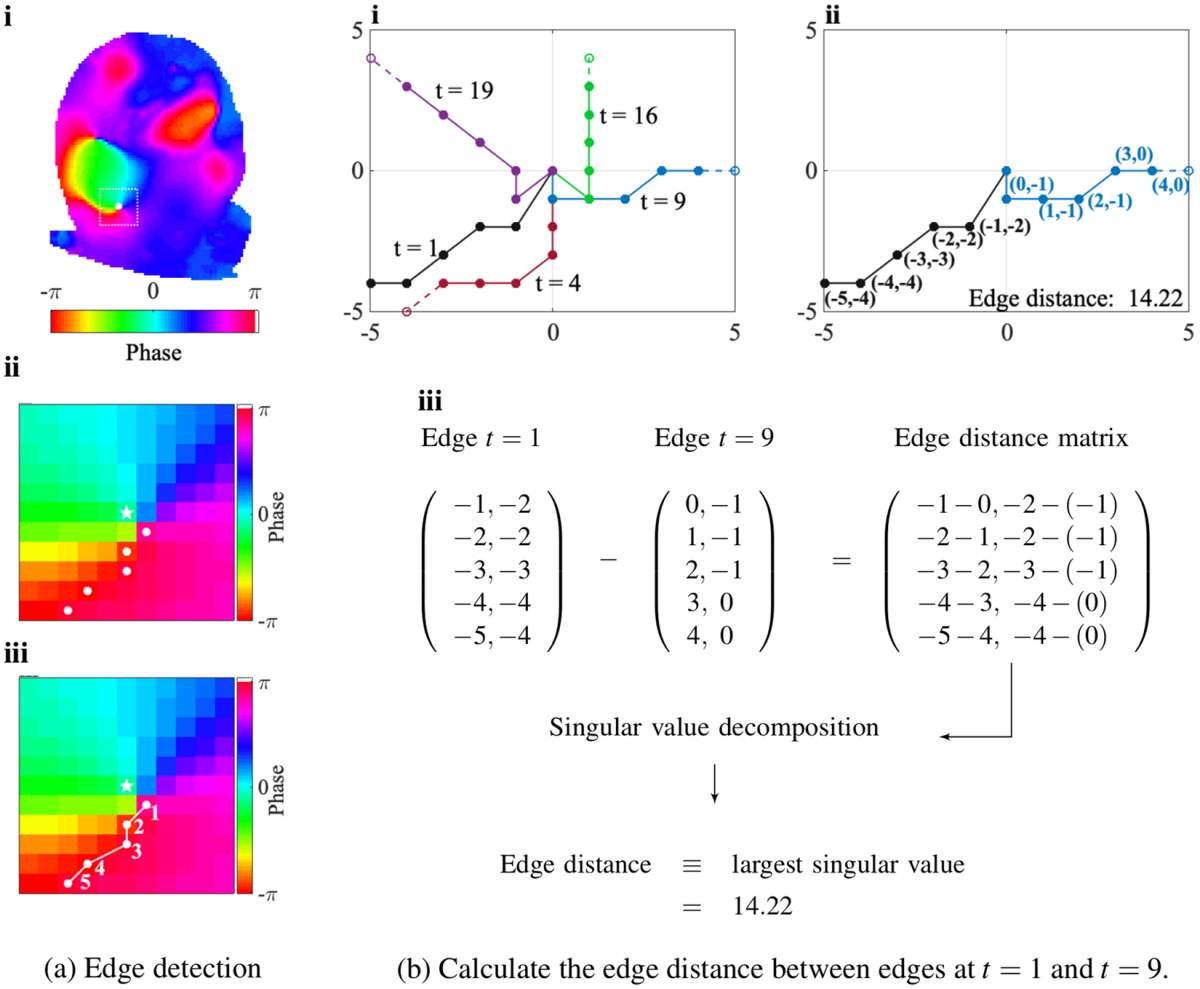
With edge pixels indexed, edge distances are calculated. In (**a**)-**i** to **iii**, the selected PS is at the center of the white box, which shows the edge detection boundary, and the raw edge pixels are indexed according to their distances to the PS. In (**b**)-**i**, the edges at different time frames are truncated to equal length to calculate edge distance. In (**b**)-**ii** to -**iii**, the calculation of distance for *t* = 1 and *t* = 9 is shown.

Edge detection is the first step in calculating the number of full rotations. Temporally an edge marks the end of the last AP and the beginning of a new AP. The leading edge of a spiral wave, is tracked by identifying regions where phase is discontinuous, jumping from *π* to −*π* rather than showing a smooth progression from *π* to −*π*, as it would from wavefront to wavetail. For the edge detection algorithm, *L* (in pixels) is defined as the length of the edge detection grid. For each time frame of the PS in Fig. 3(a)-i, edge detection was performed in a *L*×*L* square grid centred at the PS. The leading edge was detected by estimating two-dimensional derivatives using the Sobel operator^18^, yielding discrete edge pixels, which were then indexed according to their distances to the PS, as shown in Fig. 3(a)-ii to iii.

The rotation state is then determined by calculating the distance between the edges at a given time frame and the edge at time frame *t* = 1, termed the edge distance. The edge of the propagating wavefront can have differing curvatures and is not always straight and uniform, and this needs to be taken into account since rotations can only be calculated from edges of equal length. Thus, with the edges with indexed edge pixels, the longer edge is truncated to the length of the shorter edge if the two edges are of different lengths to calculate the edge distance. For example, only the first 6 pixels of the edge at *t* = 9 in Fig. 3(b)-i were used when calculating the distance. Each edge can be represented using a matrix of size *n*_*d*_-by-2, where *n*_*d*_ is the number of pixels of the edges upon truncation, and each edge matrix consists of the *x* and *y* coordinates of the edge matrices, as shown in Fig. 3(b)-ii and iii. An element-wise edge distance matrix was then calculated, the largest singular value^19^ of which was defined as the distance between the two edges. Different edge distance metrics besides the largest singular value are compared and discussed in the Supplement, Edge Distance Metrics.

**Figure 4:**
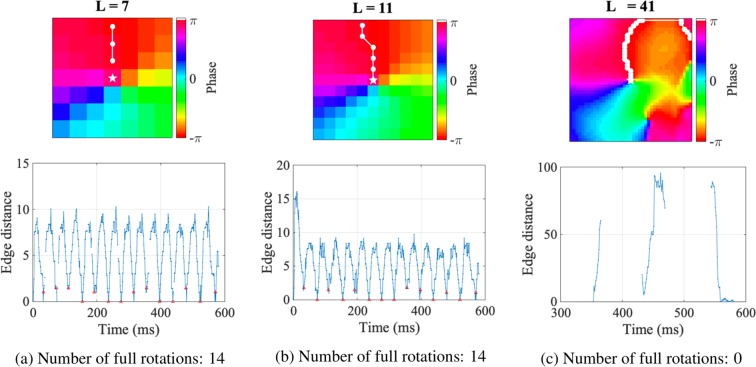
Calculation of the number of full rotations depends on the edge detection boundary *L*. (**a**–**c**): Edge distance and edge examples for different *L*. If *L* is either too small or too large, the edge distance metric may be discontinuous due to missed edge detections. If *L* is too large, points far from the PS will be detected as an edge, resulting in incorrect large values in the edge distance in (**c**).

Given all edge distances measured between the edge at *t* = 1 and later edges, the number of full rotations was estimated by calculating the number of cycles in the edge distance. In this work, cycles were determined by detecting local minima of the edge distance, marked as red triangles in Fig. 4(a–c). The examples in Fig. 4 illustrate how altering edge detection boundary *L* can provide differing rotation quantification for the same data. The examples of edge distance are shown for different edge detection boundary choices in Fig. 4. If either *L* is too small or too large, at certain time instances no edges were detected, as shown by the discontinuities in the edge distances. The presence of a large number of missing edges resulted in incomplete cycles and subsequently, incorrect estimation of the number of rotations.

## Results

### Statistics of rotational activities

For each tracked PS, different parameters, such as duration, number of rotations and spatial displacement, can be calculated to quantify the spatial and temporal stability of RDs. More importantly, by pooling all PS, group statistics may be obtained to characterise the arrhythmia behaviour. Details of the statistics we propose are given in the Supplement, Table 1.

**Figure 5:**
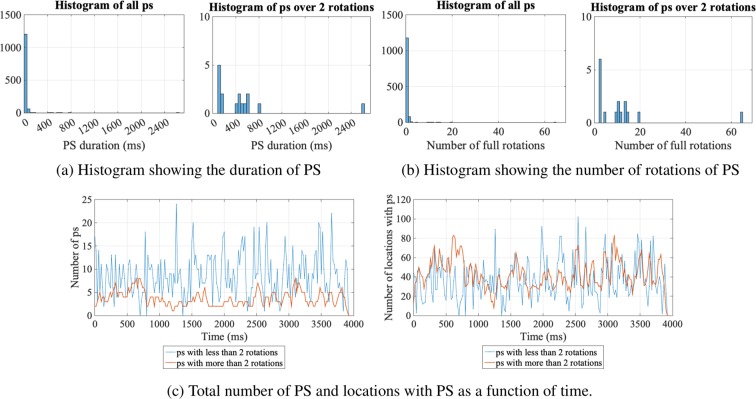
Key statistics of rotational activities include duration of PS, number of rotations of PS, total number of PS and number of locations with PS. The histograms in (**a**,**b**) show that there is a higher incidence of short-lived PS than PS with ≥2 rotations. For the PS with ≥2 rotations, most have less than 20 full rotations, and only 1 exhibits more than 60 rotations, lasting over 2400 ms. (**c**) Shows the total number of PS, as well as the number of locations with PS as a function of time for the 2 categories of PS. The number of short-lived PS is always less than that the number of PS with over 2 rotations. However, in terms of the number of locations occupied by the PS, these two categories of PS are similar.

In Fig. 5, examples of some key statistics for quantifying rotational activity properties are illustrated for a rat VF experimental model. Note that there is no consensus on the most appropriate threshold for the number of rotations for differentiating between PS and RDs, and a choice of 2 full rotations is often used as a threshold for categorising PS as RDs^20^. Thus, in our study, a threshold of 2 is adopted to define a PS as a RD. This threshold can easily be modified using the GUI in our toolbox. Figure 5(a,b) show histograms of the duration and number of rotations, respectively. Short-lived PS, i.e., those with less than 2 rotations, have a much higher incidence than those with greater than 2 rotations. As shown by the histograms for RD (i.e., PS with more than 2 rotations), most stable RDs were of a duration between 400 to 800 ms, with 10 to 20 rotations. In addition, in this example, a very stable RD was tracked, lasting over 2400 ms with more than 63 rotations. This stable RD identified by our methodology was likely to be a key contributor to sustaining fibrillation.

Figure 5(c) shows the incidence and the number of locations occupied by PS/RDs as a function of time. In this example, at each timepoint *t*, the total number of incidence and locations accumulated within a 20 ms window starting from *t* is plotted. This window length can be adjusted in the GUI input. This is one method of analysing the contribution of RDs and their stability, and thus their overall contribution to the fibrillatory mechanism over time can be determined. In this example, fibrillation is predominately driven by RDs throughout the recording.

#### Organisational level of fibrillation

**Figure 6:**
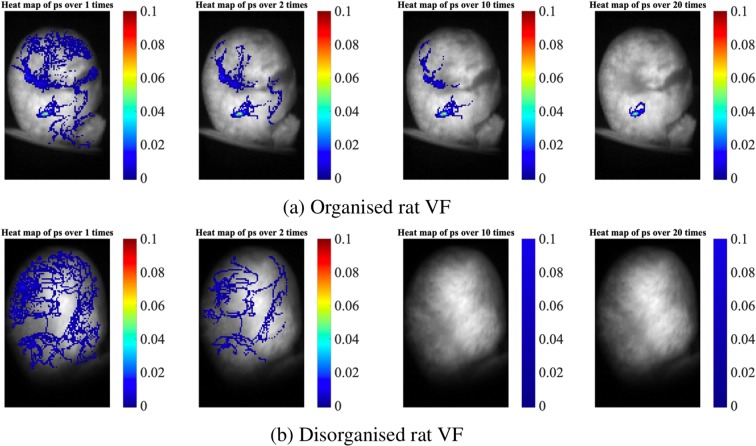
Assessing the organisation level of fibrillation quantified by PS/RD heat-maps. (**a**,**b**) Heat-maps of an organised and a disorganised example. In both examples, there are a greater number of short-lived PSs with <2 rotations than stable RDs with ≥2 rotations. Imposing a higher rotation threshold for RDs (≥10, ≥20) can help identify the most organised forms of VF, harbouring the most temporo-spatially stable RDs.

One important quantification technique we propose is the incidence of PS in the form of heat-maps, which can be used to evaluate the organisation level of fibrillation. Examples of the incidence heat-maps based on the rat VF model are shown in Fig. 6, where sub-figures (a) and (b) correspond to an organised and a disorganised example respectively. In each heat-map, the colour indicates the incidence of the PS at that pixel. For example, the yellow and red hot-spot in the figures has a value around 0.1. Since the length of the recording is 4000 ms, the duration for which this location was occupied by PSs is around 400 ms. Figure 6 also shows the results of increasing the threshold for the number of the full rotations, resulting in a more strict criteria for the stability of PS/rotational activity. In both data sets, heat-maps generated with a threshold of >1 rotations for PS are difficult to interpret, as there are a large number of areas with a high incidence of short-lived PS.

Increasing the minimum threshold for rotations to define a RD can reveal areas of interest that harbour very stable RDs with a high number of rotations that are more likely to a be involved in driving fibrillation, for example in Fig. 6(a). Conversely, where fibrillation is globally disorganised without stable RDs, a high threshold can demonstrate a lack of areas harbouring stable RDs, for example in Fig. 6(b). The rotation threshold for filtering RDs is easily modifiable in our GUI.

### Adaptability to different models

**Figure 7:**
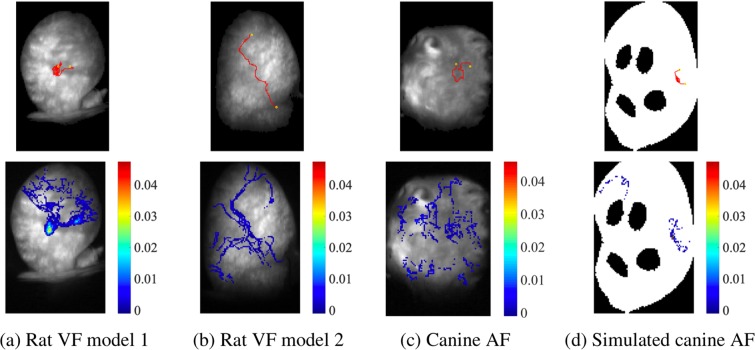
PS tracking and quantification methods applied to different AF/VF models. Sub-figures at the top are paths of the rotational activities of the longest duration and the bottom are incidence heat-maps (for RDs with ≥2 rotations) for the corresponding models. (**a**) Rat VF model 1, *N*_*r*_ = 23 and *p*_*D*_ = (8, 7); (**b**) Rat VF model 2, *N*_*r*_ = 6 and *p*_*D*_ = (21, 62); (**c**) Canine AF model, *N*_*r*_ = 5 and *p*_*D*_ = (12, 2); and (**d**) Simulated canine AF model, *N*_*r*_ = 39 and *p*_*D*_ = (5, 12).

Different experimental models are used in AF and VF studies, ranging from small and large animal models to simulated data, and thus it is important that computational methods for quantifying arrhythmia are transferable across different data sets. In Fig. 7, the reconstructed path of one RD together with the incidence heat-maps are presented using three separate fibrillation models, with sub-figures (a) to (d) corresponding to rat VF, canine AF and simulated canine AF. The proposed PS tracking and quantification methods can be applied to different AF/VF models as long as the sampling rate and background image are provided. This demonstrates the adaptability of our methodology to different fibrillation models. The top sub-figures of Fig. 7 show paths of the rotational activities of the longest duration. Constructed from the coordinates of each RD at each time frame, the RD path is a key spatial feature, which can be used to identify locations critical in driving fibrillation. Comparing the number of rotations *N*_*r*_ and the displacement of RD *p*_*D*_ across the models shows that the RDs in sub-figures (a) and (d) are more stable.

In addition to the path, the number of full rotations, *N*_*r*_, and its displacement from the beginning to end of each RD in the *x*- and *y*-axis, *p*_*D*_ = (*x*, *y*), indicate the stability of the PS. These PS features, together with global PS statistics are summarised in Table 1. In these 4 examples, the simulated canine AF example is the most organised, where the numbers of PS and RD are small, and PS and RD are constrained to a limited area. The canine AF experimental example is the most disorganised, with numerous short-lived PS meandering across a large area. These statistics, combined with the heat-maps and RD paths, may help identify critical areas for sustaining fibrillation, and have potential utility for interpreting fibrillation data in patients and guiding ablation strategy for the differing fibrillation types.

**Table 1:** Example key statistics of PS in different AF/VF models

	Rat VF 1	Rat VF 2	Canine AF	Simulated canine AF
Recording duration (ms)	4000	4000	4000	5000
Sampling rate	1000	1000	1000	200
Number of PS	888	406	2696	272
Number of PS > 2 rotations	44	21	63	48
Number of locations with PS	2111	2097	5114	381
Number of locations with PS > 2 rotations	661	605	1206	108
Maximum PS duration of PS (ms)	967	346	276	2180
Maximum number of rotations	23	6	5	39

### Impact of Spatial Resolution on Parameters Used for Quantification of Arrhythmia Characteristics

The summary statistics obtained to describe the fibrillation dynamics for a specific data set are critically dependent on the parameter choices for the analysis, such as the chosen values of *d*_*gap*_, *t*_*gap*_, and *L* at a given spatial resolution. Appropriate and consistent selection of analysis parameters is key, and differing analysis approaches between groups are thought to contribute to conflicting observations about the mechanisms of fibrillation^21^. Table 2 summarises the recommended parameters for our rat VF and canine AF experimental models. The size of the camera used in the examples shown in Figs 5–7 is 128 × 80 pixels, and the spatial resolution varies depending on the experimental model and the field of view. For the rat model, the mapped area is approximately 10 mm × 14 mm, the distance between two neighbouring pixels is approximately 0.13 mm, and for the canine model, the spatial resolution is lower and the distance between two neighbouring pixels is approximately 0.29 mm. A further detailed analysis of the impact of resolution on different parameters, namely *t*_*gap*_, *d*_*gap*_ and *L*, was undertaken and can be found in the Supplement, Sensitivity Analysis of Parameters in Response to Resolution and Supplement, Figs 1–3. Based on our analysis, the spatial resolution for reliable PS/RD quantification in optical mapping data of rat VF should be an inter-pixel distance around 0.26 mm. For spatial resolution corresponding to an inter-pixel distance of around 0.26 mm, we recommend using *d*_*gap*_ in the range of 2 to 5 pixels (which is around 0.5 to 1 mm), *t*_*gap*_ between 1 and 5 ms, and *L* of 15 pixels. Our recommended values of *d*_*gap*_ and *L* may be extrapolated to differing resolutions based on the inter-pixel distance provided, for instance by increasing *d*_*gap*_ and L by a factor of 10 (in pixels) for data acquired with a camera with 10 times the spatial resolution of ours (to correspond to the same physical distance). Similar adjustments should be made for the size of the experimental model being used to study fibrillation relative to the models studied in this paper.

**Table 2:** Parameter recommendations for quantifying rotational activity.

	Recommendations
Raw PS detection	smoothing the phase data with a 3 × 3 uniform binning
PS tracking	set the spatial gap *d*_*gap*_ as 5 pixels;
	set the temporal gap *t*_*gap*_ as 5 ms; and
	set detection boundary length *L* as 11 pixels

*These parameters are based on the spatial resolution of our mapping system for which the distance between two neighbouring pixels is in the range 0.13–0.29 mm.

## Discussion

In this paper, we demonstrated that variations in analysis parameters can dramatically affect the conclusions drawn from the analysis of fibrillation data, and that there is no universal set parameters that can be applied to all fibrillation data. We also presented a standardised framework for quantitative analysis of fibrillation data, and detailed the important principles behind the appropriate parameter selection for such analysis.

The temporal and spatial stability of RDs are important for the identification of sources that may contribute to the overall fibrillatory mechanism^20,22,23^. Hansen *et al*. proposed that the spatial stability of RDs and the percentage of AF cycles that RDs drove over a period of time was critical to their contribution to the AF mechanism^24^. In addition, there is some debate about the nature of RDs in the literature, whereby some prominent investigators have reported them as being short-lived, and temporally and spatially unstable, whilst others have reported spatial stability and anchoring to specific regions in the heart^25^. In this work, with automated RD detection, we demonstrated a methodology for tracking both stable and unstable RDs. The temporal and spatial fluctuation of their behavior can be delineated from the path tracking and quantification of RD parameters that we propose. It is likely that the conflicting observations in the literature are related to both the low resolution of clinical mapping data and the differences between methodologies for phase analysis and RD tracking. Furthermore, the importance of defining and optimising a number of parameters in identifying RDs and plotting their path have been highlighted in this paper, and we have shown how changing the spatial gap (*d*_*gap*_), temporal gap (*t*_*gap*_) and edge detection boundary length (*L*) can have a critical effect in detecting RDs and the overall interpretation of the fibrillatory mechanism.

The key limitation of our optical mapping toolbox is the sensitivity of the analysis techniques to the choices of parameters. We recommend parameter choices here which we determined for our data in parameter sensitivity studies; however, these parameters need to be decided in an experimental model specific manner by careful evaluation of outputs generated at differing thresholds as demonstrated in this study. The ability of the proposed algorithms to effectively locate and track RD will depend on the spatial resolution of the underlying data^26^. We offer recommendations here by extrapolating from our data as a starting point. Due to the low spatial resolution of many clinical mapping catheters, it is difficult to correctly identify PS from low-density electrogram data^17^. Thus, our toolbox may not be suitable for RD quantification using clinical electrograms. In addition, the presented toolbox assumes data are arranged on a regular two-dimensional spatial grid and so an interpolation step must be included in the case of a curved geometry representing three-dimensional tissue.

In conclusion, we present a comprehensive set of analytical tools and methodologies for tracking and localising RDs in myocardial fibrillation. These methodologies were able to localise sites harbouring stable RDs, to determine the differing range of fibrillatory mechanisms from the large range of data generated on RD behavioural characteristics, and are adaptable to a wide range of biological models and simulation data. We showed in detail how the choice of parameters and spatial resolution can influence detection and quantification of RDs and described the process for selecting optimal parameters. Publishing our toolbox together with the associated algorithms provides an easy and convenient off-the-shelf software platform for studying fibrillation mechanisms. Research groups with established RD analysis software can further benchmark their code against ours, to delineate the differences in analysis techniques that give rise to different conclusions.

## Experimental Details

### Recording modalities

The toolbox assumes data are recordings over time, measured on a regular 2D spatial grid (e.g. pixels). This means that each time frame of the data is a 2D image. The applications shown in this paper are either fluorescence data from an optical mapping set-up or simulated transmembrane potential data. Fibrillation data was acquired from Langendorff perfused hearts in two separate experimental models, rat VF and canine AF, and simulation data is from a canine AF model. Optical mapping data from 3D tissue are measured on a 2D surface, with signals averaged over a volume of tissue. As such, PS calculated on the 2D surface must be interpreted as arising from a 3D tissue.

### Rat ventricular fibrillation

This work was performed in accordance with standards set out in the United Kingdom Animals (Scientific Procedures) Act 1986 and was approved by the Imperial College London Ethical Review Board under the project license PEE7C76CD.

Explanted Sprague-Dawley rat hearts were heparinized, and rapidly perfused *ex-vivo* on a Langendorff apparatus with Krebs-Henseleit solution (in mmol/l: NaCl 118.5, CaCl_2_ 1.85, KCl 4.5, glucose 11.1, NaHCO_3_ 25, MgSO_4_ 2.5, NaH_2_PO_4_ 1.4) and gassed with 95% O_2_/5% CO_2_ at 37 °C ± 0.5 °C and pH 7.35 ± 0.05. A 10 minute stabilisation period was allowed during which the flow rate (10–15 ml/min), temperature (37 °C ± 0.5 °C) and perfusion pressure through the aorta was maintained between 90 and 100 mmHg. VF was induced with a burst pacing protocol (2 mA, cycle length 50–70 ms, 30 beat train). All hearts were pre-treated with a potassium channel opener, Pinacidil (30 *μ*M) during the stabilisation period described above to allow VF to be sustained for optical mapping studies. The transmembrane voltage was recorded with optical mapping using our custom made complementary metal oxide semiconductor (CMOS) camera^21,27,28^ (Cairns, Feversham UK) utilising the potentiometric dye RH237 (25 *μ*l of 1 mg/ml DMSO; Thermo-Fisher, Massachusetts, USA) and excitation contraction uncoupler blebbistatin (10 *μ*mol/L, Tocris Bio-Sciences, Cambridge UK) to eliminate motion artefacts. The mapped surface was excited at wavelength 530 nm, and emitted light was focused and filtered (>630 nm).

### Canine atrial fibrillation

These data are from our previously published study^11^. A circumflex artery-perfused isolated left atrial preparation was dissected and perfused at 37 °C with Tyrode’s solution.

Optical fluorescence data was collected with the same set up as the rat VF data. The tissue was loaded with a voltage sensitive dye (60 *μ*L of 1 mg/mL RH237) and perfused with 10 *μ*mol/L blebbistatin. Sustained AF was induced in the preparation by adding acetylcholine (10–30 *μ*M) and a burst pacing protocol.

### Simulated atrial fibrillation

The simulation data presented here are for a canine AF model, from our previous publication^11^, chosen for correspondence with the canine AF experimental model. Monodomain simulations were solved using the CARPentry simulator (available at https://carp.medunigraz.at/carputils/). Briefly, these simulations used a canine left atrial surface mesh^29^, with discretly connected two-dimensional endocardial and epicardial layers. The Ramirez–Courtemanche–Nattel canine atrial ionic model^30^ was used, with the Kneller *et al*.^31^ formulation for IKACh to incorporate the effects of acetylcholine. The model was tuned to average APD and CV values, and islands of acetylcholine release and interstitial fibrosis were incorporated as previously described^11,29^. Each of the atrial surfaces were flattened to 2D^32^, and simulated data were downsampled to a regular grid size that matched the experimental set-up.

## Supplementary information

Standardised Framework for Quantitative Analysis of Fibrillation Dynamics (Supplementary Materials)
