# Supplementary material for: Standardised Framework for Quantitative Analysis of Fibrillation Dynamics
Source: Sci Rep. 2019 Nov 13;9:16671. doi: 10.1038/s41598-019-52976-y (PMC6853901; doi:10.1038/s41598-019-52976-y)
Supplement: Supplementary file 1 — Standardised Framework for Quantitative Analysis of Fibrillation Dynamics (Supplementary Materials) [file 41598_2019_52976_MOESM1_ESM.pdf]

Standardised Framework for Quantitative Analysis of Fibrillation Dynamics (Supplementary Materials)

Xinyang Li[1,†], Caroline H Roney[2,†], Balvinder S Handa[1], Rasheda A Chowdhury[1], Steven A Niederer[2], Nicholas S Peters[1], and Fu Siong Ng[1,*]

[1]National Heart and Lung Institute, Hammersmith Campus, Imperial College London, 72 Du Cane Rd, London W12 0UQ {xinyang.li, balvinder.handa05, r.chowdhury, n.peters, f.ng}@imperial.ac.uk
[2]School of Biomedical Engineering & Imaging Sciences, King's College London, St. Thomas' Hospital, Westminster Bridge Road, UK {caroline.roney, steven.niederer}@kcl.ac.uk
[†]These authors contributed equally to this work
[*]Corresponding Author

ABSTRACT

The analysis of complex mechanisms underlying ventricular fibrillation (VF) and atrial fibrillation (AF) requires sophisticated tools for studying spatio-temporal action potential (AP) propagation dynamics. However, fibrillation analysis tools are often custom-made or proprietary, and vary between research groups. With no optimal standardised framework for analysis, results from different studies have led to disparate findings. Given the technical gap, here we present a comprehensive framework and set of principles for quantifying properties of wavefront dynamics in phase-processed data recorded during myocardial fibrillation with potentiometric dyes. Phase transformation of the fibrillatory data is particularly useful for identifying self-perpetuating spiral waves or rotational drivers (RDs) rotating around a phase singularity (PS). RDs have been implicated in sustaining fibrillation, and thus accurate localisation and quantification of RDs is crucial for understanding specific fibrillatory mechanisms. In this work, we assess how variation of analysis parameters and thresholds in the tracking of PSs and quantification of RDs could result in different interpretations of the underlying fibrillation mechanism. These techniques have been described and applied to experimental AF and VF data, and AF simulations, and examples are provided from each of these data sets to demonstrate the range of fibrillatory behaviours and adaptability of these tools. The presented methodologies are available as an open source software and offer an off-the-shelf research toolkit for quantifying and analysing fibrillatory mechanisms.

Supplement

Pre-processing of Optical Fluorescence Data

To eliminate the effects of noise present in the raw optical mapping data, spatial and temporal filters were applied. The filter type and parameter settings were selected based on our previous studies[1,2]. Specifically, data from the optical mapping set-up were imported into MATLAB using an equipment specific import function. We provide a library of import functions to bring fluorescence data from several different systems into MATLAB.

The imported data were processed using a sequence of steps as follows. First of all, fluorescence data were thresholded using a binary mask constructed from a still image taken at the beginning of the experiment to distinguish tissue from noisy pixels. Pixels were included in the analysis if they were above a threshold fluorescence using a mask constructed as a continuous domain by using imfill in MATLAB to fill any holes. The masked data were then spatially smoothed using a convolution operator (conv2 in MATLAB) to replace the value of each pixel with an average over neighbouring tissue pixels. We used a linear convolution kernel of size 3×3 pixels. For the next step, the signal from each pixel was temporally filtered using a finite impulse response filter in Matlab (Parks-McClellan algorithm; firpm), with zero-phase filtering (filtfilt) to minimise any temporal shifts caused by the filter. We used a band-pass filter with a low-band of 2Hz, and a high-band of 125% of the dominant frequency of the recording. These parameters were chosen as part of a parameter sensitivity testing study[1,2]. Baseline drift was then removed from each pixel, following[3], by subtracting a fourth order polynomial fit of the signal. Finally, the signals were normalised to the range (0, 1).

Phase Mapping

Filtered optical mapping signals were processed using a sequence of steps to calculate a zero-mean sinusoidal signal suitable for phase analysis. This was achieved using a variation of a pseudo-empirical mean decomposition technique for phase analysis[4], which we previously published[2,5]. In brief, maxima and minima of the signal were tagged using a sliding window approach. Pairs of detected maxima and minima that were below an amplitude threshold were then removed. Cubic spline fits were

performed on the maxima and minima of the signal, and the average of the maxima and minima splines was used as a moving mean signal, which was subtracted to obtain a signal of zero mean. The effects of the window length and amplitude thresholds used were investigated, and suitable values chosen to assign activation points appropriately to candidate deflections, as part of sensitivity testing of the algorithm[5]. We estimated the cycle length (CL) as the inverse of the dominant frequency of the signal. We used a window length of 90% of the estimated CL and an amplitude deletion threshold of 15% of the estimated median amplitude. Finally, the real and imaginary parts of the Hilbert transform of this zero-mean signal were plotted in the phase plane, and the angle around this trajectory gave the phase angle.

Phase maps were post-processed to identify candidate PS locations using the topological charge technique of Bray and Wikswo[6], with a contour size of 3-by-3. To increase robustness to noise, and decrease false detections, candidate PS were defined as pixels within 3 of $\pm 2\pi$. Candidate PS were only identified as true PS if they lay on a neighbourhood of three of more candidates and were the closest of their neighbours to $\pm 2\pi$. This technique was found to be more robust to noise than using a larger phase contour[1].

Statistics of Rotational Activities

To characterize the behaviour of a given rotational driver, both the spatial and temporal stability of a PS was determined. For each PS, the key statistics calculated based on PS tracking are given in Supplementary Table 1. $\bar{p}(x,y)$ and $p_{std}(x,y)$ are the average and standard deviation of the position of a given PS, respectively, and $p_D(x,y)$ measures the displacement between the beginning and end of the position of the PS. $p_{std}(x,y)$ and $p_D(x,y)$ quantify different features of the spatial stability of a PS. For a very stable PS that stays at one site all of the time, both $p_{std}(x,y)$ and $p_D(x,y)$ will be small. A PS moving within a confined area will yield relatively high $p_{std}(x,y)$ and low $p_D(x,y)$, while a PS meandering from one location to another will yield both high $p_{std}(x,y)$ and high $p_D(x,y)$. T_r and N_r are the duration and number of full rotations of a given PS, respectively, which indicate the temporal stability of the PS. The rotation frequency of the PS (f_r) was calculated from T_r and N_r.

The Group Statistic section in Supplementary Table 1 shows key statistics calculated to characterise the arrhythmia behaviour. n_{ps} and n_{ps2} are the number of all PS and PS over 2 full rotations respectively. l_{ps} and l_{ps2} are the number of locations occupied by all PS and PS over 2 full rotations, respectively. PSs with the longest duration and largest number of full rotations may represent rotational drivers that have the most dominant effect on fibrillation dynamics. As such, T_r^{max} and N_r^{max}, the maximum duration and number of full rotations of all PS respectively, are calculated. These properties are also important for global quantification of the global fibrillatory organisation. Both the PS and group statistics can be calculated and exported as .csv files by our MATLAB-based analysis toolbox.

Table 1. Key statistics of PS

Single PS Statistics	
$\bar{p}(x,y)$	The average spatial position of the PS (in pixels)
$p_{std}(x,y)$	The standard deviation of the displacement of the PS (in pixels)
$p_D(x,y)$	The average displacement from the beginning to end of the PS (in pixels)
T_r	The duration of a given PS (in ms)
N_r	The number of full rotations of a given PS
f_r	The frequency of rotation of a given PS
Group Statistics	
n_{ps}	total number of PSs
n_{ps2}	total number of PSs with 2 or more rotations
l_{ps}	total number of locations occupied by all PSs (in pixels)
l_{ps2}	total number of locations occupied by all PSs with 2 or more rotations (in pixels)
T_r^{max}	maximum duration for a single PS (in ms)
N_r^{max}	maximum number for a single PS

Sensitivity Analysis of Parameters in Response to Resolution

As discussed, the parameters d_{gap}, t_{gap} and L need to be adjusted for different experimental and simulation setups chosen for fibrillation studies. PS/RD detection may differ significantly with low spatial resolution data. To further investigate the performance of the proposed methodology under different parameter setting and spatial resolutions, sensitivity analysis was performed. To generate data of different spatial resolutions, a representative full resolution rat VF optical mapping data was downsampled by factors of 2 and 4, yielding data of 50% and 25% of the full spatial resolution.

In Supplementary Figure 1 and Supplementary Table 2, key PS/RD statistics obtained for different d_{gap} and t_{gap} with 100%, 50% and 25% of full spatial resolution are summarised. In Supplementary Figure 1, sub-panel **A** shows that the four statistics,

i.e., n_{ps}, n_{ps2}, T_r^{max} and N_r^{max}, do not differ much with t_{gap} ranging from $1ms$ to $5ms$ for any spatial resolution. As shown by sub-panel **B**, these statistics are more sensitive to changes in d_{gap}. When d_{gap} is small, PSs spatial meander within a single RD trajectory is constrained to a smaller area. Thus, a decrease in d_{gap} yields more PSs (i.e.n_{ps} and n_{ps2} increase) but shorter maximum duration and a smaller number of maximum rotations T_r^{max} and N_r^{max}. This is because trajectories are broken up into smaller trajectories that last for much fewer rotations as PSs meander between frames. The effect of d_{gap} on localisation of RDs is further illustrated in the heat maps showing RDs localisation in Supplementary Figure 2. These maps show that d_{gap} = 2 or 5 does not have a large effect on the resulting heat maps, but $d_{gap} = 1$ resulted in missed RD detection. There is always a certain degree of meander of RD. Thus, with $d_{gap} = 1$, the criteria of spatial stability for RD is too stringent, and the algorithm fails to detect any long-lived RDs for any resolutions. Figure 2 also shows that relative localisation of RD harbouring areas is preserved at 50% spatial resolution, but poor at resolutions lower than this.

Table 2. Comparing phase singularity statistics as d_{gap} and t_{gap} are varied for different spatial resolutions for a representative rat VF heart.

Resolution (%)	d_{gap}	t_{gap}	n_{ps}	n_{ps2}	l_{ps}	l_{ps2}	T_r^{max}	N_r^{max}
100	5 (0.65mm)	5 (5ms)	1256	26	2723	585	2627	63
100	2 (0.26mm)	5 (5ms)	2761	33	2723	339	419	10
100	1 (0.13mm)	5 (5ms)	8079	0	2723	0	80	1
100	5 (0.65mm)	2 (2ms)	1337	27	2723	558	2627	63
100	5 (0.65mm)	1 (1ms)	1541	27	2723	527	2627	63
50	5 (1.3mm)	5 (5ms)	366	17	753	294	1690	41
50	2 (0.52mm)	5 (5ms)	776	20	753	199	1434	34
50	1 (0.26mm)	5 (5ms)	2780	1	753	1	88	2
50	5 (1.3mm)	2 (2ms)	387	18	753	275	1690	41
50	5 (1.3mm)	1 (1ms)	438	20	753	268	1690	41
25	5 (2.6mm)	5 (5ms)	114	16	203	62	542	4
25	2 (1.04mm)	5 (5ms)	225	11	203	33	377	4
25	1 (0.52mm)	5 (5ms)	1069	0	203	0	100	1
25	5 (2.6mm)	2 (2ms)	130	16	203	57	514	4
25	5 (2.6mm)	1 (1ms)	147	14	203	57	385	4

The sensitivity of quantification to the parameter L is directly related to the cycle counting of edge distance with different L values, which is shown in Supplementary Figure **4** in the main text. In this supplementary section, more tests with different **L** values are shown in Supplementary Figure 3. With **L** ranging from 7 to 31, the minimum detection and resultant count of rotations are almost the same. When **L** is larger than 31, there are too many time frames when no edges are detected, resulting in an incorrect estimation of cycles.

In the main text, we have shown that the parameter settings we recommend also work for the canine experimental model, where the inter-pixel distance is around 0.29 mm, equating to about half of the full resolution of the rat model. However, reliable detection of rotational drivers is challenging with progressively lower spatial resolution data and prone to false positive detections[7]. This makes it difficult to extrapolate optimised d_{gap} and L values for resolutions beyond those investigated here. As shown in Supplementary Table 2 and Supplementary Figure 1, PS/RD were poorly detected and quantified with 25% of the full spatial resolution for all values of d_{gap} and t_{gap}. Based on our sensitivity analysis above, the spatial resolution required for reliable PS/RD quantification in optical mapping data of rat VF is an inter-pixel distance of approximately 0.26 mm. For spatial resolutions corresponding to an inter-pixel distance of around 0.26mm, we recommend using d_{gap} in the range of 2 to 5 pixels, t_{gap} between 1 and 5ms, and L of 15 pixels. It should be noted that at lower spatial resolutions, with a greater inter-pixel distance, the physical area subtended by a given d_{gap} will also be greater and adjustments will need to be made for this to optimise accuracy of the outputs.

We recommend a level of qualitative visual assessment of traces produced at differing edge detection lengths to ensure accurate detection of wavefronts as demonstrated in Supplementary Figure 3 before full data analysis is performed. Our recommended values of d_{gap} and L may be extrapolated to differing resolutions based on the inter-pixel distance provided, for instance by increasing d_{gap} and L by a factor of 10 (in pixels) for data acquired with a camera with 10 times the spatial resolution of ours (to correspond to the same physical distance). Similar adjustments should be made for the size of the experimental model being used to study fibrillation relative to the models studied in this paper.

Edge Distance Metrics

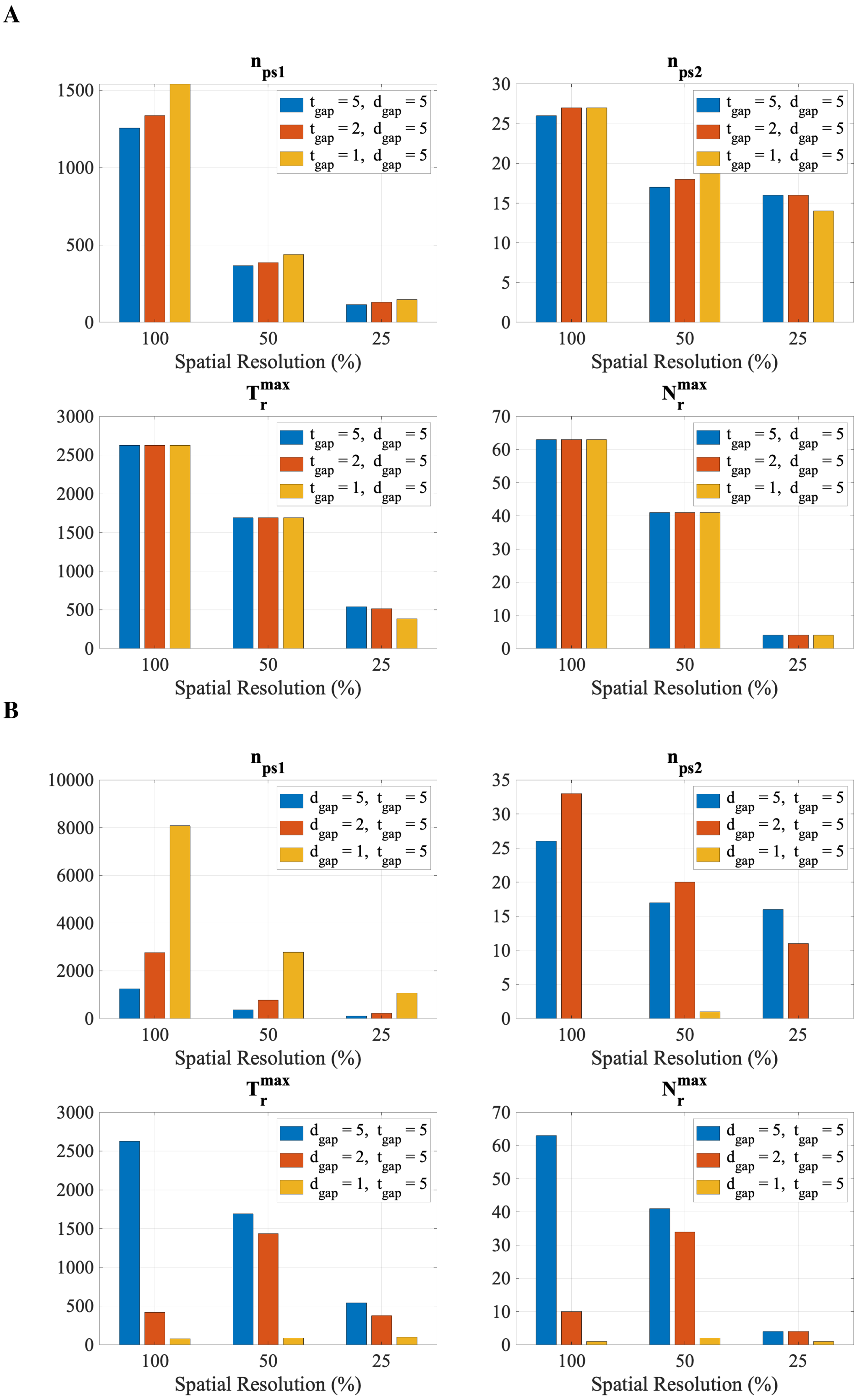

Figure 1. Comparing RD quantification for different d_{gap} and t_{gap} values and different data resolution. RD parameters n_{ps}, n_{ps2}, T_r^{max} and N_r^{max} are shown as t_{gap} is varied in (A), and as d_{gap} is varied in (B).

In this work, the largest singular value is used for edge distance calculation, which represents the l_2 norm of the edge distance matrix. We have also implemented maximum and mean Euclidean distance for comparison. As shown by Supplementary Figure 4, the cycles detected are the same with all three distance measurements with only the scale of the distances being

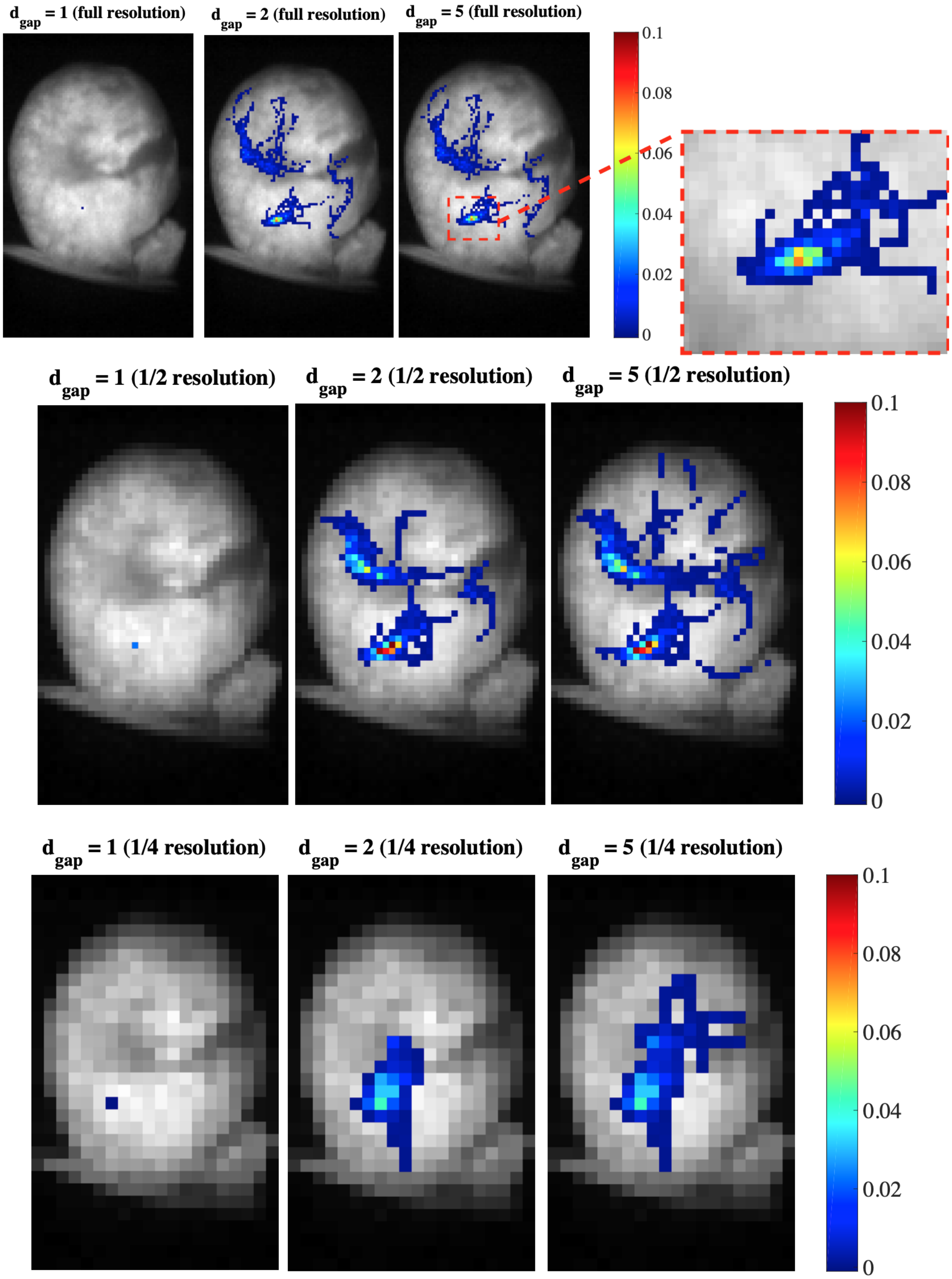

Figure 2. Representative RD heat maps of a rat VF heart comparing RD localisation for different d_{gap} and different spatial resolutions. At 50% of full spatial resolution (inter-pixel distance of 0.26mm) regions harbouring RD could be accurately localised. However, at 25% spatial resolution (inter-pixel distance of 0.52mm) the localisation is poor and inaccurate.

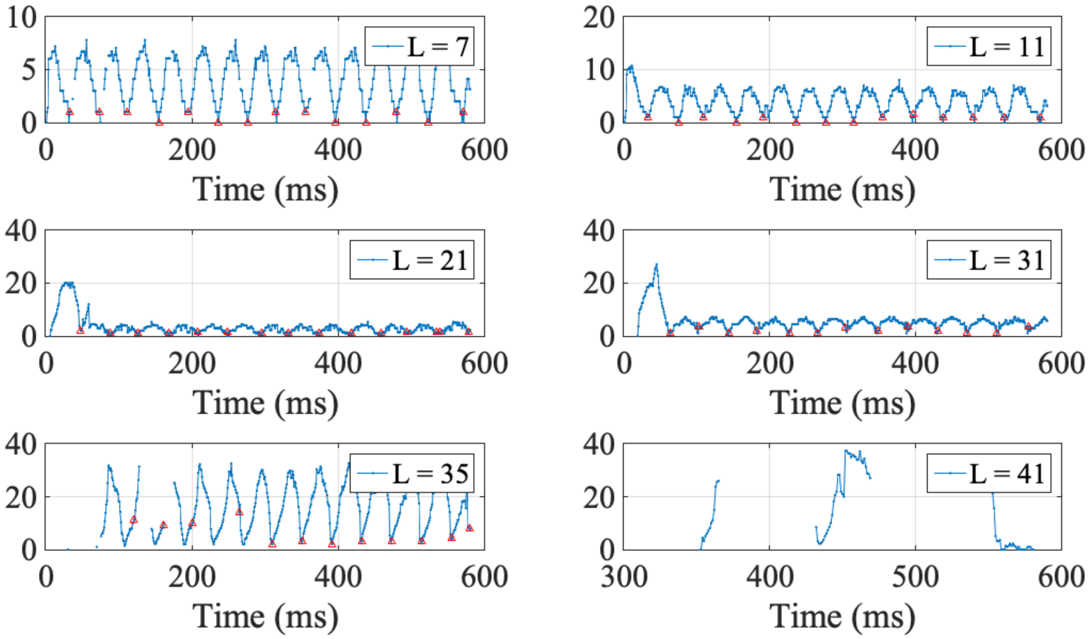

Figure 3. Edge distance traces for rotation counts for a given RD at differing L values (expressed in pixels) over time in a representative rat VF heart.

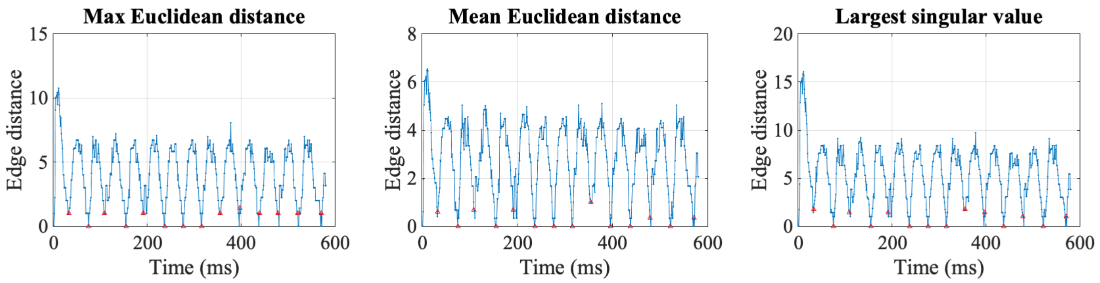

(a) Max Euclidean distance (b) Mean Euclidean distance (c) Largest singular value

Figure 4. Comparison of cycle detection using maximum and mean Euclidean distance and largest singular value. There is no differences in cycle detection between the 3 metrics.

different.

Related Methods

In addition, we have included three other commonly used mapping metrics, dominant frequency (DF), Shannon entropy (SE) and organisation index (OI), as part our open source toolbox. These are shown in Supplementary Figure 5. The methodology for calculating these metrics is well defined in the literature and previously they have been used as surrogates for localising areas harboring RDs[8]. Briefly, DF is defined as the highest peak of the frequency spectrum for each signal measured at a single site, and previous studies have measured higher DF at pivot points of rotational activities[9]. In[10], the DF frequency measure is extended to a measure of the degree of organization, termed the organizational index (OI), which is defined as the area in the power frequency spectrum under the DF peak and its harmonics divided by the total area. Entropy - for example the Shannon entropy (SE) - measures the predictability of the signal and has also been used for rotational activity mapping[11]. Note that DF and OI maps are affected by the parameter setting, such as the frequency window used to calculate the area under peaks. In this example, the frequency range to calculate DF is 20-50 Hz and a 0.75 Hz window is used to calculate the area under the DF peak and its harmonics in OI.

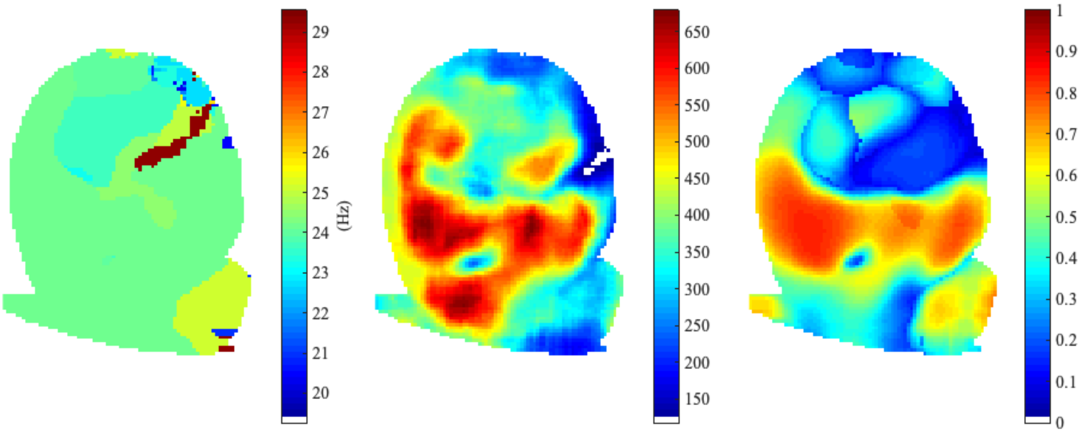

(a) DF (b) SE (c) OI

Figure 5. Application of dominant frequency (DF), Shannon entropy(SE) and organisational index (OI) analysis to rat VF data. The corresponding RD heat map generated from phase analysis is Figure 6 (a) in the manuscript. DF, SE and OI are not able to reliably locate regions harbouring RDs in this example.

References

1. Roney, C. H. *Mathematical techniques for assessing cardiac wavefront dynamics*. Ph.D. thesis, Imperial College London (2015).
2. Roney, C. H. *et al.* Determinants of new wavefront locations in cholinergic atrial fibrillation. *EP Eur.* **20**, iii3–iii15 (2018).
3. Laughner, J. I., Ng, F. S., Sulkin, M. S., Arthur, R. M. & Efimov, I. R. Processing and analysis of cardiac optical mapping data obtained with potentiometric dyes. *Am. J. Physiol. Circ. Physiol.* **303**, H753–H765 (2012).
4. Bray, M.-A. & Wikswo, J. P. Considerations in phase plane analysis for nonstationary reentrant cardiac behavior. *Phys. Rev. E* **65**, 051902 (2002).
5. Roney, C. H. *et al.* Rotor tracking using phase of electrograms recorded during atrial fibrillation. *Annals Biomed. Eng.* **45**, 910–923 (2017).
6. Bray, M.-A. & Wikswo, J. P. Use of topological charge to determine filament location and dynamics in a numerical model of scroll wave activity. *IEEE transactions on biomedical engineering* **49**, 1086–1093 (2002).
7. Roney, C. H. *et al.* Spatial resolution requirements for accurate identification of drivers of atrial fibrillation. *Circ. Arrhythmia Electrophysiol.* **10**, e004899, DOI: 10.1161/CIRCEP.116.004899 (2017).
8. Handa, B. S. *et al.* Analytical approaches for myocardial fibrillation signals. *Comput. Biol. Medicine* DOI: https://doi.org/10.1016/j.compbiomed.2018.07.008 (2018).
9. Sanders, P. *et al.* Spectral analysis identifies sites of high-frequency activity maintaining atrial fibrillation in humans. *Circulation* **112**, 798 – 797 (2005).
10. Jarman, J. W. *et al.* Organizational index mapping to identify focal sources during persistent atrial fibrillation. *J. cardiovascular electrophysiology* **25**, 355–363 (2014).
11. Annoni, E. M. *et al.* Novel quantitative analytical approaches for rotor identification and associated implications for mapping. *IEEE Transactions on Biomed. Eng.* **65**, 273–281 (2018).
